# Physical Fitness, Grit, School Attendance, and Academic Performance among Adolescents

**DOI:** 10.1155/2018/9801258

**Published:** 2018-01-15

**Authors:** Jonathan M. Cosgrove, Yen T. Chen, Darla M. Castelli

**Affiliations:** ^1^Department of Curriculum & Instruction, The University of Texas at Austin, Austin, TX, USA; ^2^Department of Kinesiology & Health Education, The University of Texas at Austin, Austin, TX, USA

## Abstract

**Objective:**

The purpose of this study was to examine the relationship of grit as a construct representing perseverance to overcoming barriers and the total number of school absences to academic performance (AP) while controlling for sociodemographics, fitness (i.e., PACER), and Body Mass Index (BMI).

**Methods:**

Adolescents (*N* = 397, SD = 1.85; 80.9% females; 77.1% Hispanic) from an urban, minority-majority city in the Southern United States completed the FitnessGram® assessment of physical fitness (e.g., aerobic capacity and Body Mass Index (BMI)) and the valid and reliable short grit survey. The schools provided sociodemographics, attendance, and AP data for the adolescents.

**Results:**

Adolescents with higher grit scores (*r*_*s*_ = 0.21,* P *< 0.001) and less total absences (*r*_*s*_ = −0.35,* P *< 0.001) performed better on AP. Hierarchical multiple regression indicated that grit and absences were associated with AP (*β* = 0.13,* P *< 0.01 and *β* = −0.35,* P *< 0.001, resp.).

**Conclusions:**

Grit and a total number of absences are significant contributors to academic success, particularly among Hispanic adolescents. Further, grit and school attendance may serve as a better measure of protective factors over proximal health measures of cardiovascular health and BMI.

## 1. Introduction

Following No Child Left Behind (NCLB), which legislated that public school monies would be tied to adequate yearly progress in academic performance (AP), it has been reported that approximately 44% of public school districts reduced resource allocation towards physical education, recess, and/or the arts in favor of increased traditional academic time and test remediation [1]. In the ensuing years, researches into the effects of physical fitness and academic achievement have shown a compelling argument for a possible relationship between the two. Furthermore, a review of literature by Tomporowski et al. [2] has proposed the importance of self-efficacious psychosocial factors in meditating this relationship. Despite this, few studies in this line of inquiry have reported actual school attendance as a confirmatory variable of their self-efficacious, amount of learning time, and effort toward AP.

### 1.1. Physical Fitness, Cognition, and Academic Performance

Adolescents who regularly participate in physical activity, including visual-motor coordination [3] and coordinated movement [4], and who are aerobically fit [5, 6] perform better academically than those youth who are sedentary. Further, health risk factors such as inflammatory biomarkers among children [7] and adiposity [8, 9] have been found to be negatively related to academic achievement. Modifiable lifestyle behaviors like healthy diet, sleep, and physical activity participation, over body weight alone, are strongly associated with academic performance [10]. Such findings suggest that school-based health interventions that focus on increasing physical activity engagement and healthy eating will likely benefit the youth, both physically and cognitively.

Physical activity interventions like Physical Activity Across the Curriculum (PAAC) and Fitness Improves Thinking (FITKids) that increase physical activity participation and subsequently improve cardiovascular fitness also increase overall academic achievement [11], executive function (Hillman, Pontifex, Castelli, Khan, Raine, Scudder, Drollette, Moore, Wu, and Kamijo, K., 2012), and cognitive control [12, 13]. Because executive function and cognitive control subserve learning, such effects are contributory to overall academic success [14], given this evidence it is justified to recommend that schools offer opportunities to be physically active before, during, and after the school day.

It is important to note that the cognitive benefits associated with physical activity and fitness are not unilateral but instead are specific to the type, timing, and intensity of physical activity. Tomporowski et al. [15] suggest that high-intensity physical activity may have the greatest gains, but those findings are not always immediately transferable to large-scale interventions across multiple schools. Further, it was also concluded that multimodal (e.g., playing games like tag) as well as aerobic physical activity (e.g., running) likely have cognitive benefits, like the PAAC and FITKids interventions. Despite the positive effects among differing modalities of physical activity intervention programs, it should be stated that the success of these programs cannot replace the learning experiences of traditional physical education or guarantee enhanced AP.

Not all interventions have equal cognitive benefits. For example, two cluster randomized controlled trials, the Active Smarter Kids (ASK) study [16] and Action Schools! BC (AS! BC) [17] failed to show an increase in academic achievement among elementary students. However, the additional time dedicated to physical activity did not result in adverse effects on academic achievement. Given the equivocal results to date, the question remains, what type, timing, and intensity of physical activity might increase the likelihood that those adolescents perform better in school.

### 1.2. Ethnicity, Socioeconomic Status, Academic Performance, and Physical Fitness

AP is dependent on a multitude of modifiable and nonmodifiable factors. Specifically, socioeconomic status (SES) and ethnicity have been identified as significant nonmodifiable inhibitors of academic success [18]. The National Center for Education Statistics has identified an achievement gap between Hispanic and White students in the public schools [19]. Considering that the 2014 U.S. census found that 50.1% of children aged 5 or under was Hispanic, narrowing the achievement gap is both timely and warranted.

The comorbidity of low SES, ethnicity, and low academic performance is complicated further as lower SES students also have an increased likelihood of health-related fitness standards falling below healthy criteria [20]. Coe and colleagues [18] found an educational and fitness disparity among 1,700 3rd, 6th, and 9th-grade students, with low SES being a proximal variable in understanding the relationship between fitness and academic performance. In the lower SES schools that might be experiencing limited access to resources and high pressure to perform academically, focusing on both educational outcomes and health issues may not be a priority. As such, one way to address ethnic differences in AP is to reduce the health risk among Hispanic children by increasing their rate of participation in physical activity [21], which has been associated with academic success [22, 23].

### 1.3. Psychosocial Determinants of Academic Success and Physical Activity Participation

Beyond nonmodifiable factors, modifiable factors like an individual perception of various psychological constructs may provide additional insight into decomposing the relationship between physical fitness and academic performance. Although over 200 studies suggest that physical fitness and to a lesser degree physical activity are positively related to cognitive performance, few studies have controlled for confounding variables such as age, ethnicity, intelligence, and psychosocial effect related to academic achievement [22]. Further, rarely did studies account for the potential of lost learning time associated with absences from school.

*Grit. *The construct of grit is defined as a “perseverance and passion towards long-term goals” [24, pg. 1]. Grit has been used as a global measure of behavior and goal acquisition that has been validated among a range of behavioral outcomes ranging from military academy matriculation, Ivy League grade point average, and national spelling bee achievement [24–26]. The underlying assumptions of grit research are that the individual displays a stable, but modifiable persistence toward achieving long-term goals. Such a disposition supports success in a wide range of endeavors. PA participation, with the long-term goal of achieving healthy fitness zone status as a proxy measure of health, could be viewed as continual work in progress requiring a degree of commitment to achieve one's desired objective. Research by Reed, Pritschet, and Cutton (2012) has demonstrated that grit is a significant predictor of moderate to vigorous PA (MVPA); however, further research is warranted to understand the relationship between grit, PA, and physical fitness.

*School Attendance. *Attending school, particularly in secondary education, remains an issue in the United States, with over 5 million school-aged children missing at least 30 days of school per year [27]. Of even more concern is when the adolescent may be absent the days leading up to the administration of academic achievement tests [28]. Missing school represents a critical loss of learning time and test preparation. Although attending school alone is not a representation of engagement in learning, not being in school inhibits academic success.

Given the existing achievement gap and health disparities between White and Hispanic adolescent students, as well as the potential to address these health and educational issues through PA interventions, this study examined the relationship of these variables to AP. Specifically, the purpose of this study was to investigate the relationship of grit, as a construct representing passion and perseverance to overcome barriers, and school attendance to academic achievement and proxy health measures while controlling for school demographics and attendance.

## 2. Methods

Multiple Institutional Review Boards approved this study at the university, district, and individual school level, with each governing body requiring that each human subject and a guardian actively consent to participate in this study. Specifically, parental consent and adolescent assent were secured for each participant. An overview of the study was presented during physical education and health classes, and once consent and assent were secured, data were collected during physical education classes.

### 2.1. Participants

In this study, adolescents enrolled in secondary education (grades 7–12, *N* = 397) across one large southern school district which serves over 83,000 students in a minority-majority city were recruited for this study. The participants were recruited from 12 different public schools (six high schools and six middle schools). All the schools that agreed to participate in this study were identified by the state education association as Title 1 Schools, meaning that they had a student population receiving free or reduced lunch more than 51%; however, the school district would not grant permission for the researcher to secure individual student SES. All students in this study participated in daily physical education for 60 minutes.

### 2.2. Instruments

There were three primary data sources utilized in this study: (1) physical fitness data collected using the FitnessGram (Cooper Institute, Dallas, TX), (2) psychosocial survey data, and (3) AP and demographic data supplied by the school district. Educational measures included standardized grade-level testing and subject test scores. Demographic information included age, sex which was coded as female = 1 and male = 2, ethnicity which was coded as White = 1, Hispanic = 2, and Other = 3, number of advanced classes, and the total number of absences for semesters 1 and 2. Student fitness was measured by the valid and reliable FitnessGram (Cooper Institute, Dallas, TX) battery of physical fitness tests. These tests were administered by the physical educator of record and primary investigator at the beginning and end of the school year.

### 2.3. Academic Performance (AP)

AP was measured by averaging year-end score on state academic accountability subject test. These tests have been used as valid and reliable measures for over 5 million public school students in the southwestern United States over two decades and are designed for vertical agreement with curricular objectives across the tested age range (8–18). Raw scores were converted to a 100-point scale and divided into quintiles for analysis (0 to 59 = 1, 60 to 69 = 2, 70 to 79 = 3, 80 to 89 = 4, and 90 to 100 = 5). Separate analyses were also conducted by subject matter (i.e., English, Math, Science, and Social Studies) with the scores for each subject similarly coded as 0 to 59 = 1, 60 to 69 = 2, 70 to 79 = 3, 80 to 89 = 4, and 90 to 100 = 5. Given the differing developmental level of the participants in this study, different AP assessments were administered. Students in the 7th grade took Reading, Math, and Writing exams, while students in the 8th grade completed the Reading, Math, Science, and Social Studies tests. High school students have more flexibility about matriculation and subsequent test administration, but this cohort supplied data for subject tests for English 1 and 2, Biology, Algebra 1 and 2, and U.S. History, corresponding to which course the student was enrolled in during the semester. Semester 1 and two final subject grades (numerical score) were provided by the district, as well as data about the rigor of the scheduled courses (number of advanced placement courses enrolled, as a representation of intellect and engagement) as well as school attendance.

### 2.4. Fitness Measurement

Health-related fitness was assessed by the physical educator of record at the beginning of the academic year (August/September). Students completed the FitnessGram battery of physical fitness tests. These tests included 20 m shuttle run (PACER; cardiovascular fitness), Body Mass Index (BMI; an indirect measure of body composition), muscular strength and endurance (push-up and curl-up tests), and flexibility by shoulder mobility stretch and prone trunk extension test. Only BMI and PACER were used in the analysis for this study. These tests have established healthy criterion and sex norms [29] and are valid and reliable measures of health-related fitness (Morrow, Martin, and Jackson, 2010). Student scores were reported as raw values (i.e., PACER was scored by the number of laps completed).

### 2.5. Grit Measurement

The 8-item Short Grit Scale is an 8-item inventory that assesses commitment and likelihood to complete long-term goals [24]. The participants were asked to self-assess the degree to which the statement was like them (e.g., setbacks (delays or obstacles) do not discourage me. I bounce back from disappointments faster than most people). The answer was on a scale ranging from 1 (i.e., not like me at all) to 5 (i.e., very much like me), with higher scores reflecting positive grit (*α* = 0.78). The survey had previously been validated to correlate positively with achievement across a wide range of constructs: marriage duration, military academy ranking, PA adherence and fitness [30], and AP in K-12 students [26].

### 2.6. Procedures

After obtaining site permission, the scope and purpose of the study were presented to students during physical education classes. Students completed surveys during class and had the primary investigator and other researchers available to answer any questions about the survey items. The physical education teacher conducted fitness testing during the first and last months of the academic year, each teacher was familiar with conducting this assessment and had received district training about how to do so. Academic accountability testing was completed in January (mid-year), March, and April. The achievement test scores, grades, sociodemographics, and school attendance data were not released by the school district until the beginning of the next school year (August).

### 2.7. Data Analysis

Continuous variables were represented by mean and standard deviations (i.e., age, BMI, PACER, grit, the total number of absences, and AP), while categorical variables are presented as absolute and relative frequencies (i.e., sex, race/ethnicity, and number of advanced classes). Spearman's correlations were conducted as an exploratory analysis to examine bivariate associations between the variables. A hierarchical multiple regression was calculated to examine the relationships between grit as well as the total number absences to AP controlling for sociodemographics, cardiovascular fitness, and BMI. To further explore the relationships between grit as well as the total number of absences to academic performance by different subjects (i.e., English, Math, Science, and Social Studies), additional hierarchical multiple regression analyses were performed individually for the four subjects. This analysis was included to provide greater specificity for possible interventions by subject matter in the future. The level of confidence was set at a 0.05 significance level. All statistical analyses were performed using SPSS version 24.0 (Chicago, IL, USA).

## 3. Results

Descriptive statistics are shown in Table 1. The participants in the present study were 397 middle school and high school students (M_age_ = 14.23, SD = 1.85). Most participants were female (*n* = 321, 80.9%). The sample comprised 306 (77.1%) Hispanic, 32 (8.1%) White, and 59 (14.8%) Black, and other race/ethnicity. These values are representative of the district at-large, as the city was considered a minority-majority community. One hundred and twenty-eight students (32.2%) were not enrolled in any advanced classes, 37 (9.3%) students took one advanced class, 73 (18.4) students took two advanced classes, 113 (28.5%) students took three advanced classes, and 46 (11.6%) students took four or more advanced classes. Results from Spearman's correlations showed that age (*r*_*s*_ = −0.23, *P* < 0.001), number of advanced classes (*r*_*s*_ = 0.38, *P* < 0.001), and BMI (*r*_*s*_ = −0.24, *P* < 0.001) as well as grit (*r*_*s*_ = 0.21, *P* < 0.001) and total number of absences (*r*_*s*_ = −0.35, *P* < 0.001) were correlated with AP. Furthermore, students who had higher grit and lower total absences performed better on English (*r*_*s*_ = 0.21 and −0.28, resp., both *P* < 0.001), Math (*r*_*s*_ = 0.16 and −0.35, resp., both *P* < 0.001), Science (*r*_*s*_ = 0.18 and −0.30, resp., both *P* < 0.001), and Social Studies (*r*_*s*_ = 0.19 and −0.23, resp., both *P* < 0.001) (see Table 2).

Next, a hierarchical regression analysis was used to examine the relationship of grit and total number of absences to AP while controlling for sociodemographics and cardiovascular fitness (i.e., PACER) and BMI. To better understand the role of grit and its relation to AP among adolescents, grit was included in all three models. In the first model of hierarchical multiple regression, sociodemographics including age, sex, ethnicity (i.e., Hispanic, Black, and Others, while White serves as a reference group), the number of advanced classes, and grit were explaining 25% of the variance in grades, *R*^2^ = 0.25, *F*(6,270) = 14.28, *P* < 0.001. Number of advance classes and grit were associated with AP (*β* = 0.36, *P* < 0.001 and *β* = 0.12, *P* < 0.01, resp.). After entering BMI and PACER in the second model, the total variance explained by the model was 26%, *R*^2^ = 0.26, *F*(8,270) = 11.53, *P* < 0.001. In the second model, BMI and PACER were not associated with AP. However, grit was related to AP (*β* = 0.13, *P* < 0.05). Finally, after entering a total number of absences with grit in the third model, the total variance explained by the model increased to 37%, *R*^2^ = 0.37, *F*(9,270) = 16.78, *P* < 0.001. In the third model, grit and total number of absences were associated with AP (*β* = 0.13, *P* < 0.01 and *β* = −0.35, *P* < 0.001, resp.); see Table 3.

Because grit and the total number of absences were significantly associated with AP, the researchers further conducted additional hierarchical multiple regressions to evaluate the relationships between grit and total number of absences to four different subjects (i.e., English, Math, Science, and Social Studies). Again, grit was included in all three models. First, the combination of sociodemographics, the number of advanced classes, and grit were explaining 26% of the variance in English, *R*^2^ = 0.26, *F*(6,293) = 16.84, *P* < 0.001. Second, BMI, PACER, and grit were explaining 27% of the variance in English, with *R*^2^ = 0.27, *F*(8,293) = 13.27, *P* < 0.001. The results showed that BMI and PACER were not associated with English. However, grit was associated with English (*β* = 0.11, *P* < 0.05). Lastly, grit and a total number of absences were explaining 34% of the variance in English, *R*^2^ = 0.34, *F*(9,293) = 16.11, *P* < 0.001. The results revealed that both grit and the total number of absences were significant predictors of English (*β* = 0.11, *P* < 0.05 and *β* = −0.27, *P* < 0.001, resp.) when all covariates are controlled (see Table 4). For other three subjects, grit was not associated with Math, Science, and Social Studies. However, the total number of absences was negatively associated with Math (*β* = −0.36, *P* < 0.001), Science (*β* = −0.20, *P* < 0.001), and Social Studies (*β* = −0.18, *P* < 0.01) after controlling for age, gender, ethnicity, the number of advanced classes, BMI, and PACER.

## 4. Discussion

Based on previous research findings, an underlying assumption of this research project was that the construct physical fitness, as a proxy measure of health, would positively impact academic achievement. This study also sought to understand the potential influence of grit and school attendance in the relationship between physical fitness and AP. The construct of grit, which has yet to be included as a psychosocial variable that might influence AP among adolescents, was selected because of its previously reported independent connections to positive fitness and academic outcomes. The generalizability of previous research was inhibited because the studies failed to account for how students persevered in their pursuit of long-term academic success as well as how often they attended school. All previous studies assumed that the student had attended school regularly and was equally engaged in the learning process, thus leading to achievement.

High levels of grit significantly predicted AP, but when this model was decomposed by subject matter, grit and attendance only predicted performance in English. This finding brings into question the preponderance of correlational analyses between physical activity/physical fitness and academic achievement. Although all of the students in this present study spoke English as the primary language, the findings are reasonable since many students may need grit to master the English language, when Spanish might be the language that was spoken in the household. This study sheds light on the possible ethnic and cultural differences in the physical activity/fitness and academic achievement line of research. We recommend that for investigations centered on academic performance to be rigorous, the studies should include measures of student attendance and/or engagement in the curriculum as well as grit. Further study on diverse populations is also warranted.

Unexpectedly grit did not predict whether someone would achieve the healthy fitness zone in fitness testing. While these findings could suggest that grit is unimportant to physical fitness in adolescents, it is possible that student agency for academic performance supersedes that of physical fitness in low SES schools that devote greater resources towards developing students who can pass accountability measures, graduate, and ensure that state monies are distributed as expected. As such, the disconnect between fidelity towards long-term goals (grit) and fitness may be a point of intervention that physical educators can target in their lesson design to facilitate this connection in the minds of students. However, a limitation of this study was that student agency for and the saliency of physical fitness as a goal was never assessed.

### 4.1. Grit and Physical Education

Previous research suggested that because physical education class is a place where students set fitness and physical activity goals as well as a course that all children are required to take over multiple years, the construct of grit can be applied and increased. The mastery of motor and tactic skills, as learning outcomes of physical education, means that students try, try, and try again as part of the process of mastering specific skills to achieve their long-term goals. Providing a mastery-oriented, over the ego-oriented, environment in physical education may help the students to increase the amount and frequency of grit applied in physical activity contexts. In a survey of physical education students, researchers discovered that students believed the motivational climate within the class was related to and even was a cause of their success [31]. Further, the students had a greater feeling of satisfaction when they achieved their goals. Future research should focus on determining if participation in goal-based physical education can, in fact, increase grit as a stable, but modifiable construct.

### 4.2. Delimitations and Limitations

Like all researches, there were several delimitations and limitations of this study. A strength is the inclusion of school attendance and grit as indirect measures of student engagement. No previous study had included grit in its examination of the AP and fitness jointly. The large sample size of Hispanic students is also a strength of this study. As scholars, we have to make inferences that research findings apply unilaterally across many different ethnicities. All students in this study participated in daily physical education for 60 minutes. The lack of direct measures of health and engagement limited this study. The researchers did not directly observe the degree of school engagement of each participant in this study. Also, although state academic accountability tests are designed to align with grade-level learning outcomes and validated by millions of students annually different grade-level students took different tests, and our measure of AP loses its predictive power for specific content areas in favor of a broader accountability to pass grade-level assessment.

### 4.3. Implications

Common assertions about the relationship between physical activity and physical fitness and how it may be physical and cognitively benefit Hispanic adolescents may be different than what was previously concluded. For Hispanic adolescents who participate in daily physical education and receive free and reduced lunch, grit and attending school are more appropriate determinants of academic success than physical fitness, the number of advanced classes that they take, or past academic success.

For adolescents to reach learning outcomes as evidence of developmental progress and school effectiveness, adolescents must attend school, given the significant relationship with academic achievement. Further study is warranted regarding how adolescents participation in physical activity may increase grit and therefore success in school.

## Figures and Tables

**Table 1:** Descriptive data for all variables.

	Total sample (*N* = 397)
Age in years, M (SD)	14.23 (1.85)
Female, *n* (%)	321 (80.9)
Race/ethnicity, *n* (%)	
White	32 (8.1)
Hispanic	306 (77.1)
Black and Others	59 (14.8)
Number of advanced classes (%)	
0	128 (32.2)
1	37 (9.3)
2	73 (18.4)
3	113 (28.5)
4 or more	46 (11.6)
BMI, M (SD)	23.82 (5.72)
PACER in laps, M (SD)	30.46 (21.0)
Number of absences, M (SD)	7.71 (8.56)
Grit, M (SD)	26.20 (4.03)
Academic performance^a^, M (SD)	3.90 (0.82)
English, M (SD)	3.89 (0.96)
Math, M (SD)	3.78 (0.94)
Science, M (SD)	3.87 (0.98)
Social studies, M (SD)	3.81 (1.02)

*Note*. ^a^Average year-end score on state academic accountability subject test and classify it into 0 to 59 = 1, 60 to 69 = 2, 70 to 79 = 3, 80 to 89 = 4, and 90 to 100 = 5.

**Table 2:** Spearman's correlations among study variables.

	(1)	(2)	(3)	(4)	(5)	(6)	(7)	(8)	(9)	(10)	(11)	(12)
(1) Age	–											
(2) Sex	0.16^*∗∗*^	–										
(3) Advanced class	−0.31^*∗∗*^	−0.15^*∗∗*^	–									
(4) BMI	0.30^*∗∗*^	0.05	−0.18^*∗∗*^	–								
(5) PACER	0.20^*∗∗*^	0.11	−0.16^*∗∗*^	−0.25^*∗∗*^	–							
(6) Absences	0.26^*∗∗*^	0.13^*∗*^	−0.25^*∗∗*^	0.15^*∗*^	−0.03	–						
(7) Grit	−0.11^*∗*^	0.01	0.12^*∗*^	−0.01	0.03	−0.07	–					
(8) AP	−0.23^*∗∗*^	−0.10	0.38^*∗∗*^	−0.24^*∗∗*^	−0.09	−0.35^*∗∗*^	0.21^*∗∗*^	–				
(9) English	−0.22^*∗∗*^	−0.12^*∗∗*^	0.38^*∗∗*^	−0.21^*∗∗*^	−0.13^*∗*^	−0.28^*∗∗*^	0.21^*∗∗*^	0.81^*∗∗*^	–			
(10) Math	−0.19^*∗∗*^	−0.08	0.33^*∗∗*^	−0.17^*∗∗*^	−0.12^*∗*^	−0.35^*∗∗*^	0.16^*∗∗*^	0.75^*∗∗*^	0.59^*∗∗*^	–		
(11) Science	−0.22^*∗∗*^	−0.07	0.35^*∗∗*^	−0.19^*∗∗*^	−0.05	−0.30^*∗∗*^	0.18^*∗∗*^	0.77^*∗∗*^	0.59^*∗∗*^	0.59^*∗∗*^	–	
(12) Social studies	−0.27^*∗∗*^	−0.09	0.33^*∗∗*^	−0.20^*∗∗*^	−0.06	−0.23^*∗∗*^	0.19^*∗∗*^	0.71^*∗∗*^	0.61^*∗∗*^	0.49^*∗∗*^	0.52^*∗∗*^	–

*Note*. AP = academic performance; ^*∗*^*P* < 0.05. ^*∗∗*^*P* < 0.01.

**Table 3:** Summary of hierarchical regression analysis for variables predicting academic performance (*N* = 397).

Predictor variable	Model 1	Model 2	Model 3
Age	−0.02	0.02	0.09
Sex			
Male	−0.15^*∗*^	−0.14^*∗*^	−0.14^*∗*^
Race/ethnicity			
Hispanic	−0.31^*∗∗*^	−0.30^*∗∗*^	−0.27^*∗∗*^
Black and Others	−0.06	−0.05	−0.05
Number of advanced classes	0.36^*∗∗*^	0.35^*∗∗*^	0.27^*∗∗*^
BMI		−0.11	−0.09
PACER		−0.10	−0.11
Number of absences			−0.35^*∗∗*^
Grit	0.12^*∗*^	0.13^*∗*^	0.13^*∗∗*^
*R*^2^	0.25	0.26	0.37
Δ*R*^2^		0.01	0.11

*Note*. ^*∗*^*P* < 0.05. ^*∗∗*^*P* < 0.01.

**Table 4:** Summary of hierarchical regression analysis for variables predicting English (*N* = 397).

Predictor variable	Model 1	Model 2	Model 3
Age	−0.03	0.01	0.05
Sex			
Male	−0.22^*∗*^	−0.20^*∗∗*^	−0.20^*∗*^
Race/ethnicity			
Hispanic	−0.26^*∗∗*^	−0.25^*∗∗*^	−0.23^*∗∗*^
Black and Others	−0.03	−0.03	−0.03
Number of advanced classes	0.38^*∗∗*^	0.36^*∗∗*^	0.31^*∗∗*^
BMI		−0.10	−0.07
PACER		−0.10	−0.10
Number of absences			−0.27^*∗∗*^
Grit	0.10	0.11^*∗*^	0.11^*∗*^
*R*^2^	0.26	0.27	0.34
Δ*R*^2^		0.01	0.07

*Note*. ^*∗*^*P* < 0.05. ^*∗∗*^*P* < 0.01.
